# The phylogeny and distribution of *Wolbachia* in two pathogen vector insects, Asian citrus psyllid and Longan psyllid

**DOI:** 10.3389/fcimb.2023.1121186

**Published:** 2023-03-06

**Authors:** Da Ou, Jun-Hong Qiu, Zheng-Qin Su, Lei Wang, Bao-Li Qiu

**Affiliations:** ^1^ Chongqing Key Laboratory of Vector Insects, College of Life Sciences, Chongqing Normal University, Chongqing, China; ^2^ Guangdong Laboratory for Lingnan Modern Agriculture, Guangzhou, China; ^3^ Engineering Research Centre of Biological Control, Ministry of Education, South China Agricultural University, Guangzhou, China

**Keywords:** Cornegenapsylla sinica, Diaphorina citri, Wolbachia, pathogen vector, phylogeny, localization pattern

## Abstract

**Background:**

*Wolbachia* is the most abundant bacterial endosymbiont among insects. It can play a prominent role in the development, reproduction and immunity of its given insect host. To date, *Wolbachia* presence is well studied within aphids, whiteflies and planthoppers, but relatively few studies have investigated its presence in psyllids.

**Methods:**

Here, the infection status of *Wolbachia* in five species of psyllid, including Asian citrus psyllid *Diaphorina citri* and longan psyllid *Cornegenapsylla sinica* was investigated. The phylogenetic relationships of different *Wolbachia* lines and their infection density and patterns in *D. citri* and *C. sinica* from different countries was also examined.

**Results:**

The infection rates of *Wolbachia* in *D. citri* and *C. sinica* were both 100%, and their sequencing types are ST173 and ST532 respectively. Phylogenetic analysis revealed that the *Wolbachia* lines in *D. citri* and *C. sinica* both belong to the Con subgroup of *Wolbachia* supergroup B. In addition, *Wolbachia* displayed a scattered localization pattern in the 5th instar nymphs and in the reproductive organs of both *D. citri* and *C. sinica* but differed in other tissues; it was highest in the midgut, lowest in the salivary glands and medium in both the testes and ovaries.

**Conclusion:**

Our findings assist in further understanding the coevolution of *Wolbachia* and its psyllid hosts. Given that *Wolbachia* could play an important role in insect pest control and pathogen transmission inhibition, our findings may also provide new insights for development of control strategies for *D. citri* and *C. sinica*.

## Introduction

Symbiotic bacteria are ubiquitous in animal hosts, among which the endosymbiont *Wolbachia* is the most abundant one in arthropods (Dale et al., 2006; Zug and Hammerstein, 2012). *Wolbachia* contains several supergroups, all of which are different in their physiological roles and host distribution (Lo et al., 2007; Ros et al., 2009; Bing et al., 2014). In arthropod hosts, *Wolbachia* has been reported in various tissues but mainly resides in the reproductive organs, where it is associated with the induction of different reproductive alterations such as feminization, parthenogenesis, male killing, and cytoplasmic incompatibility (Stouthamer et al., 1999; Hancock et al., 2011; Lv et al., 2021), in turn, aiding the spread of *Wolbachia* infection in its host populations (Saridaki and Bourtzis, 2010). Recently, extensive evidence has shown that *Wolbachia* can benefit a number of insects *via* a mutualistic relationship (Zug and Hammerstein, 2015). For example, *Wolbachia* can protect arthropod hosts against a variety of pathogens and abiotic stresses (Teixeira et al., 2008; Brownlie et al., 2009; Iturbe-Ormaetxe et al., 2011). Some *Wolbachia* strains are also essential for successful egg development, such as in bed bugs, parasitic wasps and collembolan species (Dedeine et al., 2001; Timmermans and Ellers, 2008; Kremer et al., 2009; Hosokawa et al., 2010), while others can enhance the fecundity of its female host insect (Dedeine et al., 2001; Dobson et al., 2004; Fry et al., 2004; Dong et al., 2006).

The infection, distribution of *Wolbachia* and its ability to manipulate the reproductive properties of arthropod hosts have attracted much interest concerning its role in the host’s biology, ecology, and evolution, as well as in the development of novel, symbiont based and environmentally friendly based methods for pest and disease management (Bourtzis and Miller, 2006; Hedges et al., 2008; Moreira et al., 2009; Laidoudi et al., 2020; Ilinsky et al., 2022; Zong et al., 2022). For instance, recent studies have shown that, the presence of *Wolbachia* in some insect species may provide antiviral protection, and inhibit the infection and transmission of certain pathogens such as Dengue, Zika, Chikungunya, Yellow fever, Mayaro viruses and rice ragged stunt virus (RRSV) (Moreira et al., 2009; Walker et al., 2011; Van den Hurk et al., 2012; Frentiu et al., 2014; Tan et al., 2017; Ryan et al., 2019).

The Asian citrus psyllid *Diaphorina citri* Kuwayama and longan psyllid *Cornegenapsylla sinica* Yang et Li are both phloem feeding insect pests. *D. citri* is considered one of the most destructive citrus pests due to its capability to transmit the bacterial causal agent of Huanglongbing or citrus greening, *Candidatus* Liberibacter asiaticus (*C*Las) (Halbert and Manjunath, 2004), while *C. sinica* is a devastating pest of Longan that vectors the longan pathogen witches’ broom virus (LgWB) (Chen et al., 1992; Seo et al., 2017; Tran et al., 2019). New environmentally friendly strategies and products are urgently required to manage these pests since few efficient control strategies are available due to the rapid evolution of high levels of insecticide resistance (Cuthbertson and Vanninen, 2015; Chen et al., 2021).

Similar to many other insect species, *D. citri* and *C. sinica* are also infected with *Wolbachia* (designated *w*Di and *w*Sin). Although the direct influence of *Wolbachia* on *D. citri* and *C. sinica* biology remains to be determined, recent studies indicate that the relative abundance of *w*Di may be associated with the abundance of *C*Las within hosts (Fagen et al., 2012), where it may contribute to the regulation of phage lytic cycle genes in *C*Las (Jain et al., 2017). These findings highlight the importance of *w*Di in citrus greening disease management.

As previously mentioned, strategies based on the maternally inherited endosymbiont *Wolbachia* is currently under development for insect borne diseases control by either population replacement or population suppression strategies (Hoffmann et al., 2011; Zheng et al., 2019; Crawford et al., 2020; Neupane et al., 2022). Such disease control approaches are based on the ability of *Wolbachia* to inhibit the pathogen transmission of insect vectors (Hedges et al., 2008; Moreira et al., 2009; Bian et al., 2013). Thus, to achieve this purpose, the *Wolbachia* infection and distribution status in these psyllid insects should be determined.

In the current study, the adults of *D. citri, C. sinica* as well as another three similarly geospatially distributed species of psyllid in South China, *Macrohomotoma sinica* Yang et Li, *Blastopsylla occidentalis* Taylor and *Pseudophacopteron canarium* Yang et Li, were collected. The infection and distribution of the *Wolbachia* endosymbiont in these five species, and their phylogenetic relationship with each other was investigated. This was expected to provide new insights for the development of alternative and environmentally friendly strategies for insect vector control.

## Materials and methods

### Insect collecting and rearing

The five populations of psyllid, *D. citri*, *C. sinica*, *M. sinica*, *B. occidentalis* and *P. canarium* were collected from citrus, longan, banyan, eucalyptus and olive trees respectively during August 2022. They were continuously reared on their respective seedling plants in separated glasshouses in South China Agricultural University, Guangzhou at 26-28°C, 60-80% relative humidity and 14:10h (L:D) photoperiod. New seedling plants with fresh shoots were provided for the sample cultures every two weeks.

### PCR detection of *Wolbachia*

Total DNA was extracted from each single adult of the five species of psyllid using the TIANamp Genomic DNA Kit (Tiangen, Beijing, China) following the manufacturer’s instructions. The general primers used for *Wolbachia* detection were *wsp* 81F and *wsp* 691R (Zhou et al., 1998), which target a DNA fragment of *Wolbachia*’s outer surface protein (*wsp*) gene (**Table 1**). The PCR reactions were run in a 25μl buffer containing 1μl of the template DNA lysate, 1μl of each primer, 2.5mM MgCl_2_, 200mM for each dNTP and 1 unit of DNA Taq polymerase (Invitrogen, Guangzhou, China). PCR products were visualized by 1.0% agarose gel electrophoresis, stained with Gold View in 0.5 × TBE buffer (Sangon, Shanghai, China) and photographed under UV light. In total, DNA from 100, 100, 64, 52 and 22 individual adults of *D. citri*, *C. sinica*, *M. sinica*, *B. occidentalis* and *P. canarium* was extracted respectively.

**Table 1:** The primers used for *wsp* and MLST gene amplification of Asian citrus psyllid and longan psyllid.

Gene	Primer sequence (5′-3′)	Size range (bp)	References
*wsp*	wsp81-F: 5’- TGGTCCAATAAGTGATGAAGAAAC-3’	600	Zhou et al., 1998
	wsp691-R: 5’- AAAAATTAAACGCTACTCCA-3’		
*gatB*	gatB-F: 5’- GAKTTAAAYCGYGCAGGBGTT-3’	471	Baldo et al., 2006
	gatB-R: 5’- TGGYAAYTCRGGYAAAGATGA-3’		
*coxA*	coxA-F: 5’- TTGGRGCRATYAACTTTATAG-3’	487	Baldo et al., 2006
	coxA-R: 5’- CTAAAGACTTTKACRCCAGT-3’		
*hcpA*	hcpA-F: 5’- GAAATARCAGTTGCTGCAAA-3’	515	Baldo et al., 2006
	hcpA-R: 5’- GAAAGTYRAGCAAGYTCTG-3’		
*ftsZ*	ftsZ-F: 5’- ATYATGGARCATATAAARGATAG-3’	524	Baldo et al., 2006
	ftsZ-R: 5’- TCRAGYAATGGATTRGATAT-3’ -3’		
*fbpA*	fbpA-F: 5’- GCTGCTCCRCTTGGYWTGAT-3’	509	Baldo et al., 2006
	fbpA-R: 5’- CCRCCAGARAAAAYYACTATTC-3’		

### Gene sequencing of *mtCOI* and *Wolbachia* MLST

The total template DNA extraction, PCR amplification reaction and target DNA visualization of psyllid adults was the same as previously described for the PCR detection of *Wolbachia*. The primers used for the PCR amplification of psyllid *mtCOI* gene are COI F: 5’ AGGAGGTGGAGACCCAATCT 3’ and COI R: 5’ TCAATTGGGGGAGAGTTTTG 3’ (Boykin et al., 2012). Target PCR products were purified and sent out for complete bidirectional sequencing in Sangon Biotech Co., Ltd. (Shanghai, China).

For the MLST analysis of *Wolbachia* in *D. citri* and *C. sinica*, the *wsp* gene and five MLST genes (*gatB*, *coxA*, *hcpA*, *ftsZ*, and *fbpA*) were amplified *via* the special PCR primers shown in **Table 1**. Again, the target PCR products were purified and sent to Sangon Biotech Co., Ltd. for complete bidirectional sequencing.

### Phylogeny analysis of the psyllid species

The *mtCOI* gene sequences from another 14 psyllid species were selected as references based on the study of Percy et al. (2018) (**Table 2**). The *mtCOI* sequences were firstly aligned using Lasergene v7.1 (DNASTAR, Inc., Madison, WI), and then the phylogeny of the *mtCOI* sequences were analyzed independently with Neighbor-Joining Algorithm (NJ) based on the Tamura-Nei model using MEGA 6.0 software. The *mtCOI* sequence of the bed bug *Pariaconus ohiacola* (KY294009) was used as the out group (**Table 2**). A discrete gamma distribution was applied for each analysis with 1,000 bootstrap replicates (NJ BS).

**Table 2:** The reference sequences of mtCOI genes used in the phylogenetic analysis.

Location	Host	Isolate	Accession number
Guangzhou, China	*Cornegenapsylla sinica*	GZCS	MN728680
Saga, Japan	*Cacopsylla chinensis*	I-1mc	AB720877
Taiwan, China	*Cacopsylla chinensis*	JA-1	AB364024
Taiwan, China	*Cacopsylla chinensis*	JB	AB364027
Taiwan, China	*Cacopsylla qianli*	MA	AB364033
Taiwan, China	*Cacopsylla qianli*	MA-Q	AB364034
Taiwan, China	*Cacopsylla qianli*	MB-Q	AB364035
Ibaraki, Japen	*Cacopsylla pyrisuga*	Cp-2fs	AB721007
Ibaraki, Japen	*Cacopsylla pyrisuga*	Cp-3ms	AB721008
Ibaraki, Japen	*Cacopsylla pyrisuga*	Cp-5fs	AB721009
Guangzhou, China	*Diaphorina citri*	GZCP	MF614818
My Tho, Vietnam	*Diaphorina citri*	psy57-5	FJ190382
Fujian, China	*Diaphorina citri*	psy52-4	FJ190365
Florida, USA	*Diaphorina citri*	psy56-5	FJ190377
Taiwan, China	*Diaphorina communis*	DcomMH	MG988724
Taiwan, China	*Diaphorina lycii*	DP1	MF426267
Hawaii, USA	*Pariaconus ohiacola*	OC_Hi51	KY294009

### Sequence typing and phylogenetic analysis of *Wolbachia*

The *wsp* and five MLST genes of *Wolbachia* strains in the *D. citri* (*w*Di) and *C. sinica* (*w*Sin) were blasted in the GenBank on the NCBI website. The phylogenetic relationships of *Wolbachia* in these two vector psyllids was analyzed based on their *wsp* and the five MLST genes. Firstly, the *wsp* genes of *Wolbachia* from 8 subgroups of supergroup A and 7 subgroups of supergroup B were used as references. The *wsp* gene of *Wolbachia* from the filarial parasite *Brugia malayi* (AJ252061) was used as the out group in the phylogeny analysis based on the *wsp* (**Table 3**). Secondly, the *wsp* gene sequences of *Wolbachia* obtained in this study were separately compared with other sequences from China, Thailand, Singapore, Pakistan, Iran, India, Saudi Arabia, Jamaica, Brazil and USA strains that are deposited in both the NCBI and the *Wolbachia* database (http://pubmlst.org/wolbachia/wsp/) (**Table 4**), using the Bayesian methods as described above. The MLST genes of *Wolbachia* from 5 subgroups of supergroup A and 3 subgroups of supergroup B were used as references; the MLST gene sequences of *Cimex lectularius* were used as the out group in the phylogeny analysis of *Wolbachia* based on the MLST genes (**Table 5**).

**Table 3:** The reference sequences of *Wolbachia wsp* genes used in the phylogenetic analysis.

Supergroup	Subgroup	Host	GenBank accession number
A	Pap	*Phlebotomus papatasi*	AF020082
	Aus	*Glossina austeni*	AF020077
	Ri	*Drsophila simulans*	AF020070
	Mel	*Drsophila melanogaster*	AF020063
	AlbA	*Aedes albopictus*	AF020058
	Uni	*Muscidifurax uniraptor*	AF020071
	Kue	*Ephestia kuehnlella*	AF071911
	MorS	*Glossina morsitans*	AF020078
B	Con	*Tribolium confusum*	AF020083
	Stri	*Laodelphax striatellus*	AF020080
	Dei	*Trichogramma deion*	AF020084
	Kay	*Trichogramma kaykai*	AF071924
	Div	*Apoanagyrus diversicornis*	AF071916
	Pip	*Aedes albopictus*	AF020059
	Pip	*Culex pipiens*	AF020061
F	——	*Brugia malayi*	AJ252061

**Table 4:** Accession numbers for *wsp* gene sequences obtained from GenBank and the *Wolbachia* database.

Location	Host	Isolate	Accession number
Guangzhou, China	*Cornegenapsylla sinica*	GZCS	OP902290
Guangzhou, China	*Diaphorina citri*	GZCP	OP902291
Beihai, China	*Diaphorina citri*	wCitri Beihai	GQ385974
FuZhou, China	*Diaphorina citri*	wCitri FuZhou	GU480071
Shenzhen, China	*Diaphorina citri*	wCitri Shenzhen	GU480072
Thailand and Singapore	*Diaphorina citri*	Co-1	160^a^
Guilan, Iran	*Diaphorina citri*	FD2	KC539848
Sargodha, Pakistan	*Diaphorina citri*	Pakistan-1	MN809922
Raju, India	*Diaphorina citri*	DC	MK303765
Makkah, Saudi Arabia	*Diaphorina citri*	20.025-3	OP131602
Jizan, Saudi Arabia	*Diaphorina citri*	21.05-1	OP131599
Ribeirão Preto, Brazil	*Diaphorina citri*	Dcit_B_wDc01	294^a^
Jamaica and Caribbean	*Diaphorina citri*	L118	KX198666
Florida, USA	*Diaphorina citri*	FloridaWsp_2	OP131600
Florida, USA	*Diaphorina citri*	FloridaWsp_1	OP131601
Outgroup	*Brugia malayi*	–	AJ252061

a Code numbers in the Wolbachia database (pubmlst.org).

**Table 5:** The reference sequences of *Wolbachia* MLST genes used in the phylogenetic analysis.

ID	Supergroup	Host	MLST genes					
			ST	*gatB*	*coxA*	*hcpA*	*ftsZ*	*fbpA*
1	A	*Drosophila melanogaster*	1	1	1	1	1	1
12	A	*Aedes albopictus*	2	3	2	2	10	3
13	A	*Ephestia kuehniella*	19	7	6	7	3	8
5	A	*Drosophila bifasciata*	34	14	15	16	13	15
68	A	*Agelenopsis aperta*	65	32	33	38	30	37
268	B	*Diaphorina citri*	174	9	91	109	15	27
269	B	*Diaphorina citri*	175	109	86	88	126	27
343	B	*Diaphorina citri*	225	140	66	29	112	27
356	B	*Diaphorina citri*	236	167	91	170	126	27
1810	B	*Diaphorina citri*	461	246	11	29	209	4
1811	B	*Diaphorina citri*	462	106	11	106	208	162
1812	B	*Diaphorina citri*	463	109	86	101	81	27
23	B	*Acraea eponina*	4	12	12	13	2	22
19	B	*Chelymorpha alternans*	7	9	14	15	12	14
33	B	*Encarsia formosa*	18	17	18	20	15	18
24	B	*Gryllus firmus*	21	15	16	17	16	16
34	B	*Nasonia vitripennis*	26	9	8	9	7	9
32	B	*Ostrinia scapulalis*	27	9	9	10	8	10
39	B	*Lycaeides idas*	36	9	36	40	7	9
69	B	*Polistes dominulus*	37	9	9	6	8	10
99	B	*Horaga onyx*	39	12	14	13	2	41
26	B	*Drosophila simulans*	15	5	4	5	4	6
27	B	*Drosophila simulans*	16	5	4	4	4	5
20	B	*Tribolium confusum*	30	6	5	6	18	7
25	B	*Teleogryllus taiwanemma*	32	9	25	30	20	25
100	B	*Surendra vivarna*	40	38	38	29	35	42
87	B	*Drosophila innubila*	98	79	71	88	69	27
92	B	*Polybia* sp.	103	69	65	87	62	27
620	B	*Bemisia tabaci*	378	105	88	106	7	387
36	F	*Cimex lectularius*	8	26	27	31	24	28

The phylogeny of *wsp* and MLST sequences was analyzed independently *via* Neighbor-Joining Algorithm (NJ) based on the Tamura-Nei model using MEGA 6.0 software. A discrete gamma distribution was applied for each analysis with 1,000 bootstrap replicates. For *Wolbachia* genes, unique sequences were searched for in the *Wolbachia* MLST database (http://www.pubmlst.org/wolbachia/), resulting in their ST numbers being determined (Baldo et al., 2006).

### Infection density of *Wolbachia* in *D. citri* and *C. sinica*

The density of *Wolbachia* in different *D. citri* and *C. sinica* tissues was determined using qPCR. The psyllid adults were dissected at 7 days post-emergence after the final molt under a stereomicroscope, which included their salivary glands, midgut, testes, and ovaries. Primers used in *Wolbachia* quantitative detection were 16S-F: 5’-GAGTGAAGAAGGCCTTTGGG-3’, and 16S-R 5’-CACGGAGTTAGCCAGGACTTC-3’ (Gong et al., 2020), which amplify a fragment of the *Wolbachia 16S rRNA* gene. The *Actin* genes of *D. citri* (forward: 5’-ACTGCCCTGGCTCCCTC T-3’, reverse: 5’-CGGACTCGTCGTATTCTTGTTT-3’) and *C. sinica* (forward: 5’-ACTGCCCTG GCTCCCTCT-3’, reverse: 5’-CGGACTCGTCGTATTCTTGTTT-3’) were used as the housekeeping genes. Three repeats and 5 adult individuals in each repeat were detected.

### FISH visualization of *Wolbachia*

*Wolbachia* distribution differs in nymphal and adult tissues. Fluorescence *in situ* hybridization (FISH) was used according to the description in Li et al. (2020). Briefly, entire 4^th^ instar nymphs, and the ovaries and testes of 5-7d old adults after dissection, were fixed for 30min in fresh 4% paraformaldehyde prepared in 1×PBS with 0.1% Triton X-100. The samples were then washed three times for 5min in 1×PBS, immersed in hybridization solution overnight in a 46˚C water bath in the dark. Following this, the samples were washed once for 5min in each of four solutions in turn (2×SSC with 0.015% (w/v) DTT; 1×SSC with 0.015% (w/v) DTT; 0.5×SSC with 0.015% (w/v) DTT; 1×PBS alone), then stained for 1h with VECTASHIELD^®^ Antifade Mounting Medium with DAPI (Vector Laboratories, CA, USA) at room temperature. They were then washed again in 1×PBS before being mounted with anti-fluorescence quenching mounting medium. The distribution was then be observed under an inverted fluorescence microscope (Nikon Eclipse TieU). The Cy5 5’-end-labeled *Wolbachia 16S rRNA* probes (W2-Cy3: 5’-CTTCTGTGAGTACCGTCATTATC-3’) were used for the hybridization.

### Statistical analysis

The relative titers of *Wolbachia* in the different treatments were firstly normalized and then calculated using the method of 2^-ΔΔct^ with the accompanying software in a Bio-Rad thermocycler (Bio Rad CFX Manager). All data was analyzed using one-way analysis of variance (ANOVA), and means were compared using the Duncan’s test (SPSS 17.0) at *P*<0.01. All figures were drawn using Sigmaplot 10.0.

## Results

### *Wolbachia* infection rates in different species of psyllids

**Figure 1:**
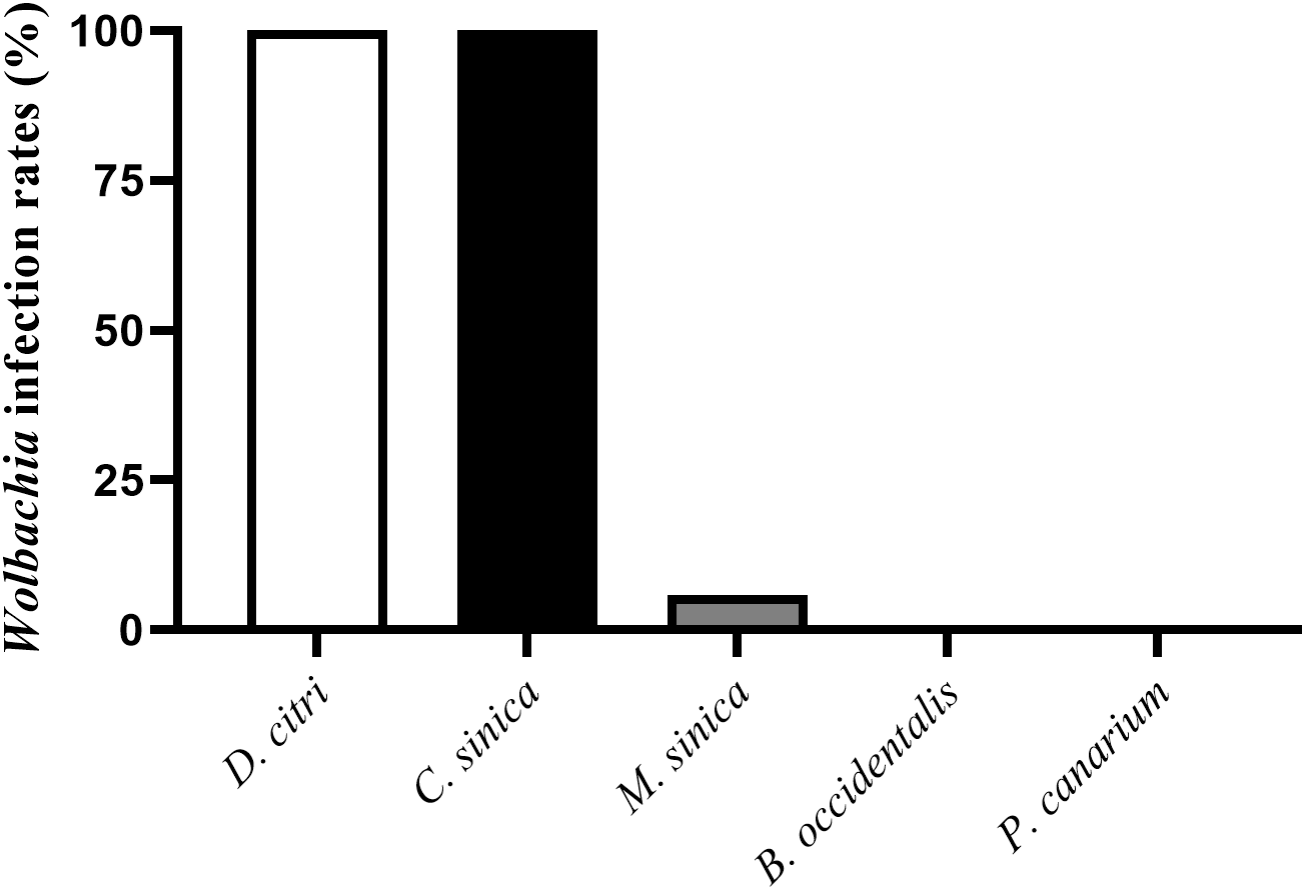
*Wolbachia* infection rates in different species of psyllids.

The PCR detection results revealed that *Wolbachia* was present in *D. citri*, *C. sinica* and *M. sinica* adults, with its infection rates being 100% in both *D. citri* (100/100) and *C. sinica* (100/100), and approximately 6.25% in *M. sinica* (4/64). However, *Wolbachia* was absent in *B. occidentalis* (0/52) and *P. canarium* (0/22) (**Figure 1**).

### Phylogenetic relationships of *D. citri* and *C. sinica*

**Figure 2:**
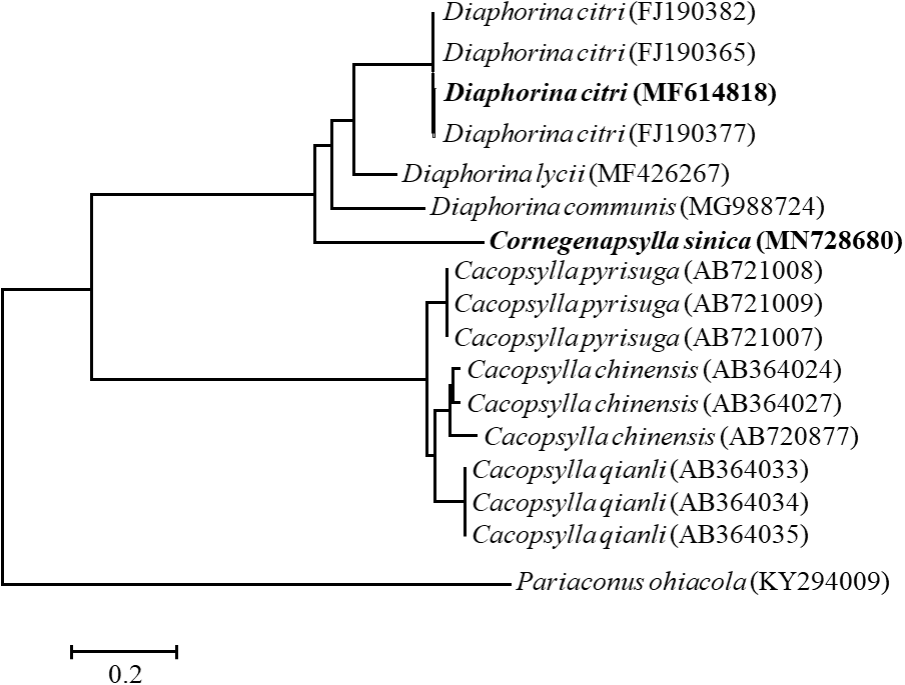
The phylogenetic relationship of *Diaphorina citri* and *Cornegenapsylla sinica* based on *mtCOI* gene. The tree was constructed and analyzed by Neighbor-Joining (NJ) method using 1000 bootstraps replicates. Numbers at the nodes indicate the percentages of reliability of each branch of the tree. Branch length is drawn proportional to the estimated sequence divergence.

The *mtCOI* gene of *D. citri* and *C. sinica* were successfully amplified, with the sequences submitted to GenBank (accession number MF614818 for *D. citri*; MN728680 for *C. sinica*). The phylogeny trees of *D. citri*, *C. sinica* and the 14 other *Diaphorina* insects showed that the phylogeny trees of *D. citri*, *C. sinica* and the 14 other psyllids showed that *D. citri* and *C. sinica* were firstly clustered into one branch, and the *D. citri* and *C. sinica* clustered with the *Cacopsylla* psyllids into one peripheral branch (**Figure 2**). As expected, *D. citri* and *C. sinica* have a close phylogenetic relationship to each other.

### Sequence typing and phylogenetic relationships of *Wolbachia* in *D. citri* and *C. sinica*

**Figure 3:**
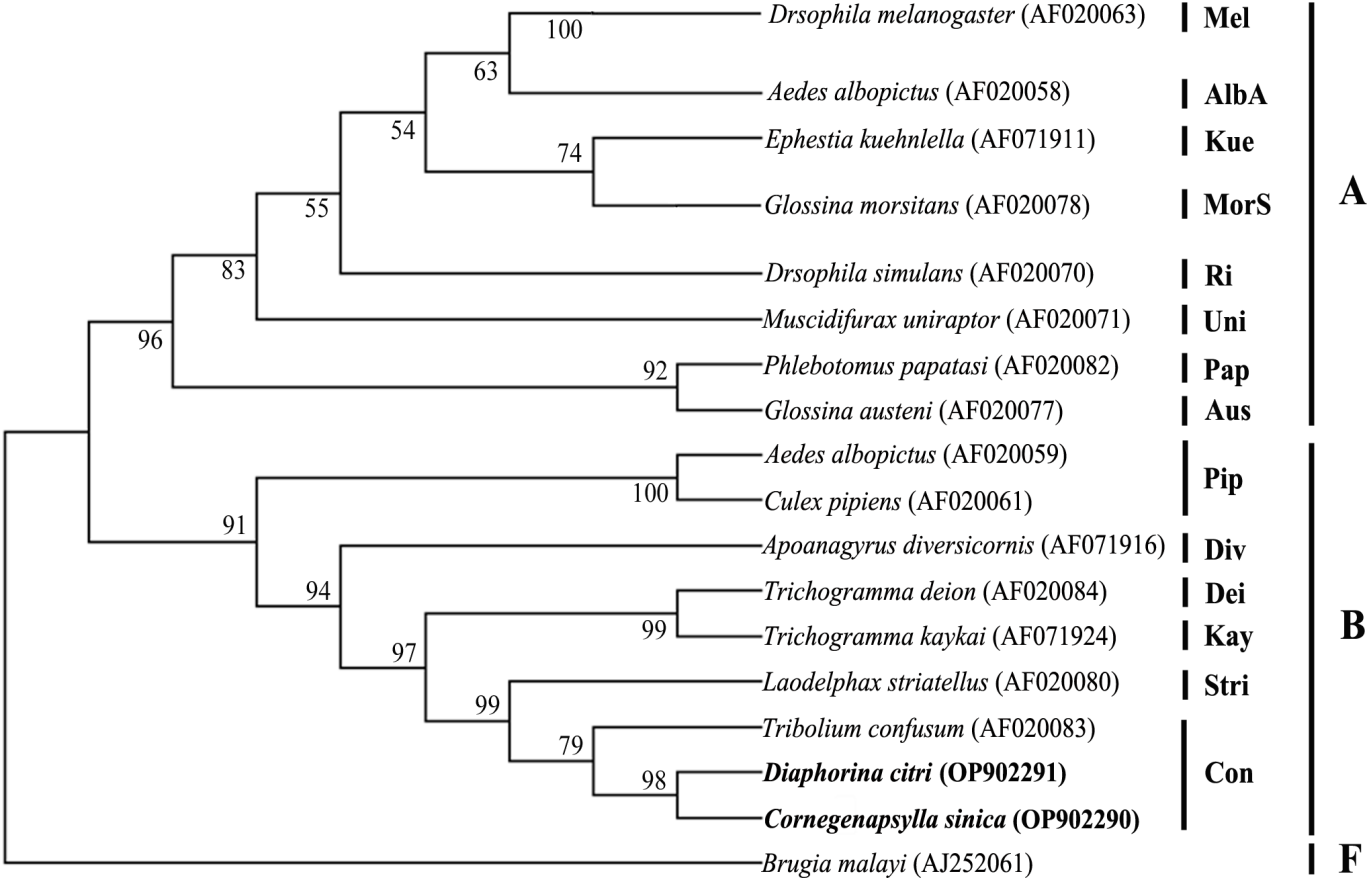
The phylogenetic relationships of *Wolbachia* from different insect hosts based on the DNA sequence of *wsp* gene. The tree was constructed and analyzed by Neighbor-Joining (NJ) method using 1000 bootstraps replicates.

The results from the sequence typing analysis revealed that the *Wolbachia* in *D. citri* and *C. sinica* were ST173 and ST532 respectively (**Table 6**). The phylogenetic analysis of *Wolbachia* based on their *wsp* genes showed that all the *Wolbachia* lines were clustered into two main branches, i.e., A branch and B branch; *Wolbachia* lines from *D. citri* and *C. sinica* in the current study were firstly clustered into one branch of *Con* subgroup with *Tribolium confusum*, then clustered with *Trichogramma deion* (*Dei* subgroup) and *Trichogramma kaykai* (*Kay* subgroup), *Apoanagyrus diversicornis* (*Div* subgroup), *Aedes albopictus* and *Culex pipiens* (*Pip* subgroup) in turn; all of which belong to the supergroup B (**Figure 3**).

**Table 6:** The PCR outputs of *Wolbachia* MLST and *wsp* genes in *C. sinica and D. citri*.

ID	Host	MLST genes						*wsp*				
		ST	*gatB*	*coxA*	*hcpA*	*ftsZ*	*fbpA*	*wsp*	HVR1	HVR2	HVR3	HVR4
267	*D. citri*	173	109	86	29	81	27	160	2	17	3	23
1936	*C. sinica*	532	158	4	282	7	6		159	35	3	

**Figure 4:**
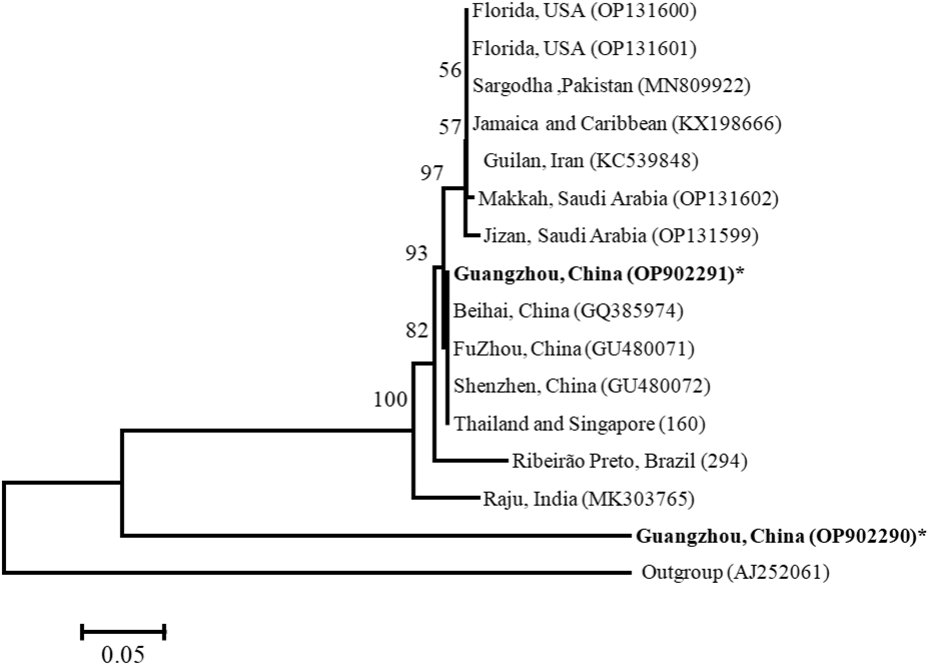
Bayesian analysis of *Wolbachia* in *Diaphorina citri* and *Cornegenapsylla sinica* host Guangzhou (China) populations, compared with those available in GenBank and the *Wolbachia* database based on *wsp* gene. The tree was constructed and analyzed by Neighbor-Joining (NJ) method using 1000 bootstraps replicates. Refer to **Figure 2** for the meaning of the numbers and branch length.

**Figure 5:**
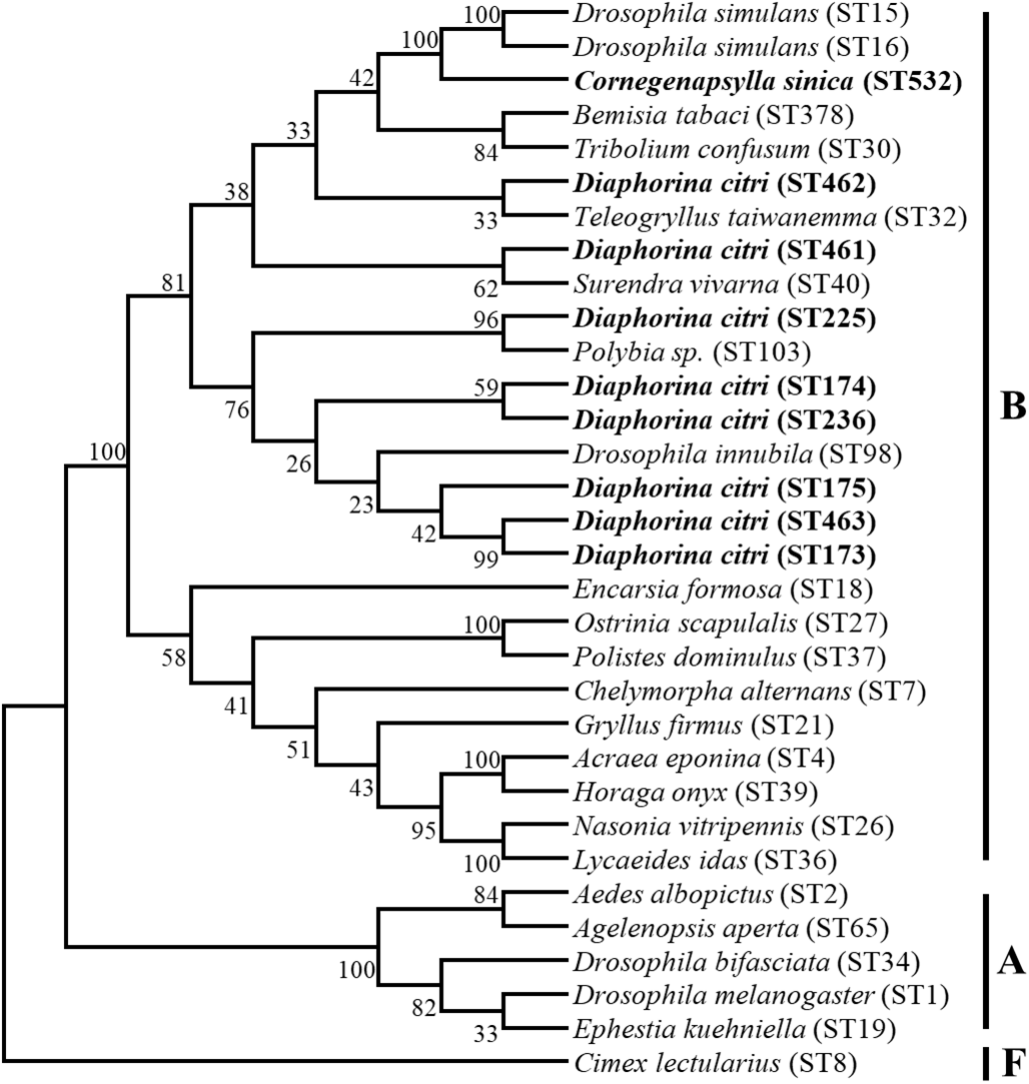
The phylogenetic relationships of *Wolbachia* from different insect hosts based on the DNA sequence of MLST gene. The tree was constructed and analyzed by Neighbor-Joining (NJ) method using 1000 bootstraps replicates.

Our Bayesian phylogenetic analysis of the *Wolbachia* indicated that all the *Wolbachia* strains in the *D. citri* populations collected from different regions in China were highly homologous; clustering into one branch. There were small variants to the strains of *D. citri* populations collected from South Asia, West Asia and America in another sister branch. Although *Wolbachia w*Sin in *C. sinica* also belongs to the *Wolbachia* supergroup B, it is phylogenetically distant from all the *Wolbachia w*Di strains (**Figure 4**).When analyzed, the phylogeny of *Wolbachia* different lines based on the MLST genes, *Wolbachia* lines of *C. sinica* (ST532) and *Drosophila simulans* (ST16) were firstly clustered into one branch, then into another branch with *Wolbachia* of *D. citri* (ST173). *Wolbachia* lines of *B. tabaci* (ST378) and *T. confusum* (ST30) were firstly clustered into one branch together, becoming a sister branch of *Wolbachia* lines of *C. sinica*, *D. simulans* and *D. citri* in supergroup B (**Figure 5**). This result is consistent with the phylogeny of *Wolbachia* based on the *wsp*, *gatB*, *coxA*, *hcpA*, *ftsZ*, and *fbpA* genes (**Figures 3**, **S1**).

### Infection of *Wolbachia* in *D. citri* and *C. sinica*

**Figure 6:**
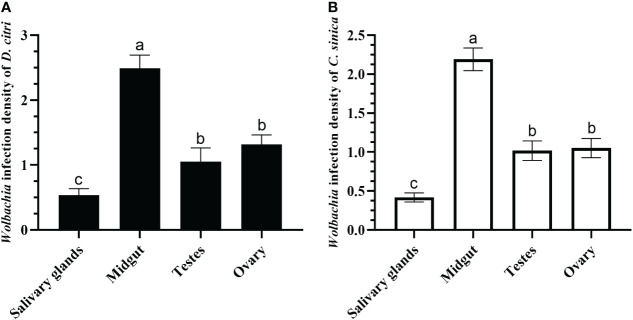
*Wolbachia* titers in salivary glands, midgut, testes, and ovary of *Diaphorina citri* **(A)** and *Cornegenapsylla sinica* **(B)**. Error bars indicate 95% confidence intervals. Different letters indicate significant differences among different tissues (*P <*0.05).

The qRT-PCR results demonstrated that the infection densities of *Wolbachia* in different tissues of *D. citri* and *C. sinica* were consistent. The infection in the midgut was highest, followed by the ovary and testes. Infection in the salivary glands was the lowest when comparing the four tissues (**Figure 6**).

### Distribution of *Wolbachia* in *D. citri* and *C. sinica*

**Figure 7:**
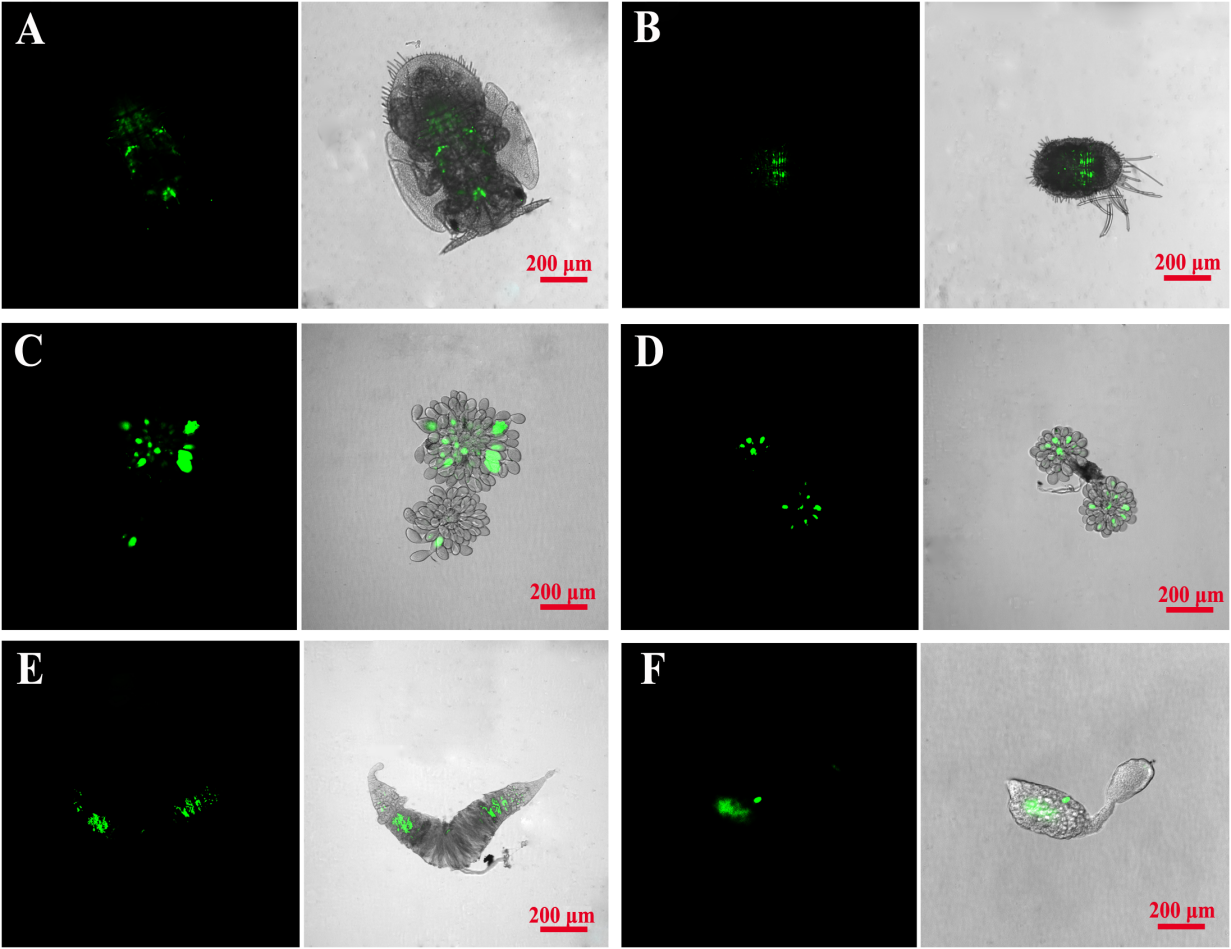
Fluorescence *in situ* hybridization visualization of *Wolbachia* in Asian citrus psyllid and longan psyllid. *Wolbachia* was respectively stained green by specific probes. **(A, B)**: *D. citri* and *C. sinica* 4^th^ nymphal stage; **(C, D)**: *D. citri* and *C. sinica* ovary; **(E, F)**: *D. citri* and *C. sinica* testes.

The localizations of *Wolbachia* in *D. citri* and *C. sinica* hosts were visualized by FISH. *Wolbachia* was clearly scattered throughout the whole body of the 5^th^ nymphal instar and the reproductive organs of adults (**Figure 7**).

## Discussion

To date, *Wolbachia* is well studied within aphids, whiteflies and planthoppers, but currently few studies have revealed the presence and physiological roles of *Wolbachia* in psyllids. This is partly due to the difficulty in getting a *Wolbachia* negative population *via* the inactivation method and at the same time not affecting other endosymbionts in the same psyllid host (Liu et al., 2020). In the current study, our results revealed that the infection rates of *Wolbachia* are 100% in *D. citri* (*C*Las vector) and *C. sinica* (LgWB vector), but much lower or even negative in the psyllids *M. sinica*, *B. occidentalis* and *P. canarium*. Interestingly, based on global samples of *Wolbachia*, Lashkari et al. (2014) discovered a strong association between the *mtCOI* gene of *D. citri* and their *Wolbachia* strains, while our results of phylogenetic analysis showed that the *Wolbachia* strains are conserved in the psyllids of Guangzhou (China) populations. Whether the infection of *Wolbachia* is related to transmit pathogens of these psyllid hosts needs to be further investigated; Understanding this could facilitate our understanding of the interaction relationship between *Wolbachia*, its vector insect and the pathogen. With regard to the different *Wolbachia* infection rates in the psyllids, we presumed that the immune-related benefits may be the determining factor. *Wolbachia* has been confirmed in having the ability to confer protection against pathogen infection in its hosts, leading to reduced pathogen load or decreased host mortality associated with pathogen infection (Hedges et al., 2008). *D. citri* (*C*Las vector) and *C. sinica* (LgWB vector) appear to require larger *Wolbachia* infection rates (Teixeira et al., 2008; Osborne et al., 2012; Chrostek et al., 2013; Stevanovic et al., 2015), with this hypothesis being evidenced by several previous studies which have revealed that *D. citri* is naturally infected by the *Wolbachia* strain *w*Di at a prevalence of 100% (Meyer and Hoy, 2008; Wang et al., 2010; Guidolin and Consoli, 2013; Chu et al., 2016).

Phylogenetic analysis of the genetic relationship between *Wolbachia* and their hosts is essential to understanding the evolutionary pathways and transmission processes of *Wolbachia* in different hosts. The vector mediated interspecific transmission of intracellular bacterial endosymbionts has been confirmed by phylogeny studies that insects sharing the same ecological niche contacts with each other can acquire different *Wolbachia* strains horizontally, such as sharing the same food sources (Oliver et al., 2010);, host plants (Caspi-Fluger et al., 2012; Li S. J et al., 2017), predators (Jaenike, 2007; Gehrer and Vorburger, 2012) and parasitoids (Duron et al., 2010; Li et al., 2011; Ahmed et al., 2015). Interestingly, Pigeault et al. (2014) reported the strain *w*Con tended to reduce reproductive investment but maintained or increased immune parameters. *Wolbachia* strains from *D. citri* and *C. sinica* in the current study were clustered into one branch of *Con* subgroup in the supergroup B, which can assist in predicting the roles of *Wolbachia* in *D. citri* and *C. sinica.*

*Wolbachia* can also be horizontally transmitted between intraspecies of insect hosts (Ahmed et al., 2013; Chu et al., 2016; Li S. J et al., 2017; Li Y. H et al., 2017; Liu et al., 2023). For example, by comparing the phylogeny of different *Bemisia* species and their endophytes, Ahmed et al. (2013) revealed the discordance of *Wolbachia* with their whitefly hosts and testified that *Wolbachia* can achieve host transfer through horizontal transmission. In the present study, we revealed the *Wolbachia* lines in *D. citri* and *C. sinica* are ST173 and ST532 based on *wsp* and MLST genes. We therefore predict that horizontal transmission of *Wolbachia* may not occur between *C. sinica* and *D. citri* since they do not share the same host plants.

By using fluorescent *in situ* hybridization with *Wolbachia* specific probes, we revealed the spatial distribution of *Wolbachia* in *D. citri* and *C. sinica*. Overall, the distribution patterns of the profiles *w*Di and *w*Sin also aligned with findings from previous work (Ren et al., 2018), which showed that *Wolbachia* was clearly scattered throughout the whole body of the 5^th^ nymphal instar and the reproductive organs of adults. The distribution patterns of endosymbionts in their hosts have significant impacts on their transmission and ability to affect their hosts (Li S. J et al., 2017). According to the conclusion of Ahmed et al. (2015), a “scattered” distribution pattern provides more chance for parasitoids to pick up the endosymbiont by their mouthparts when feeding or during a probing check before egg-laying. They then vector the horizontal transmission of the endosymbiont, such as *Wolbachia* in the current study, between different psyllid individuals.

In addition, the qRT-PCR detection revealed that the infection density of *Wolbachia* was highest in the midgut, medium in the ovaries and testes, and lowest in the salivary glands of both *D. citri* and *C. sinica*. The salivary glands are the key organ for the pathogen transmission of vector insects. Begomoviruses can even self-proliferate in the salivary glands of the whitefly *B. tabaci* (Wang et al., 2022), and *C*Las multiplication was also detected in salivary glands of *D. citri* (Wu et al., 2018). However, Fraser et al. (2020) demonstrated that there is no association between a *Wolbachia* strain’s ability to inhibit Dengue infection in the mosquito and either its typical density in the midgut or salivary glands, or the degree to which it elevates innate immune response pathways in the mosquito. Concerning the different function of *Wolbachia* in various hosts’ salivary glands, the interaction relationships between *Wolbachia* and *C*Las, *Wolbachia* and LgWB are worthy of further research.

Significant progress has been achieved in developing *Wolbachia* based strategies for the control of insect vectors. Guo et al. (2022) demonstrated that the *Aedes albopictus* HC line infected with a trio of *Wolbachia* strains exhibited almost complete blockade of Dengue virus (DENV) and Zika virus (ZIKV) in horizontal and vertical transmission. Also, *Wolbachia* strengthens host immunity, cellular regeneration and causes the expression of microRNAs which could potentially be involved in virus inhibition (Reyes et al., 2021; Yu et al., 2022). Gong et al. (2020) reported the first successful transfer of *Wolbachia* endosymbiont into a pest planthopper, and that the endosymbiont self-spreads into the host population, causes sufficiently high levels of CI, and inhibits transmission of the rice plant virus RRSV by *Nilaparvata lugens*. Importantly, it mitigated RRSV associated disease symptoms in rice plants.

When several symbionts are simultaneously present within the same host, interactions between them can take place and affect the dynamics of the microbial population. In these cases, hosts are often seen as shared limited spaces and as resources in which competition for exploitation, termed ‘the tragedy of the commons’ occurs (Vautrin and Vavre, 2009). Bacterial competition for limited resources occurs within infected *Wolbachia* populations but not in uninfected *Wolbachia* populations, therefore, implying that bacterial interactions can cause differences in pathogen infection rates among various insect populations (Vautrin and Vavre, 2009). The populations of vector insects carrying *Wolbachia* are more challenging to infect with pathogens (Krstić et al., 2018). Therefore, the strength of pathogen inhibition is considered to depend on the density of *Wolbachia* (Teixeira et al., 2008). Further research is also required to determine whether *Wolbachia* can affect LgWB spread and for its utilization in *C. sinica* control.

## Conclusions

To summarize, characterizing the diversity and ecology of *Wolbachia* may shed light on the coevolution of *Wolbachia* and its psyllid hosts, as well as the interactions between psyllid borne pathogens. In addition, to identifying the *Wolbachia* strains in *D. citri* and *C. sinica* psyllid species, findings from this work may benefit the understanding of *Wolbachia* psyllid relationships. Since *Wolbachia* induced CI could play an important role in insect pest or pathogen control strategies by reducing insect population size or acting as a drive system for disseminating desirable genes/alleles (Sinkins and Gould, 2006), the next research objectives should be the CI function identification for the ST173 *Wolbachia* line in *D. citri* and ST532 *Wolbachia* line in *C. sinica*. This would provide new insights for the development of *D. citri C*Las and *C. sinica* LgWB control strategies.

## Data availability statement

The datasets presented in this study can be found in online repositories. The names of the repository/repositories and accession number(s) can be found below: https://www.ncbi.nlm.nih.gov/, MN728680, https://www.ncbi.nlm.nih.gov/, OP902291, https://www.ncbi.nlm.nih.gov/, OP902290.

## Author contributions

DO and B-LQ, conceived and designed the experiments. DO, J-HQ, Z-QS, and LW, performed the experiments. DO, J-HQ, and Z-QS, analyzed the data. B-LQ, contributed to reagents, materials, and analysis tools. DO and B-LQ, wrote the manuscript. All authors contributed to the article and approved the submitted version.

## Supplementary material

Supplementary Figure 1 The phylogenetic relationships of *Wolbachia* from different insect hosts based on the DNA sequence of **(A)** *coxA*, **(B)** *fbpA*, **(C)** *ftsZ*, **(D)** *gatB* and **(E)** *hcpA* gene. The tree was constructed and analyzed by Neighbor-Joining (NJ) method using 1000 bootstraps replicates.
