# Supplementary figures and images for: The phylogeny and distribution of Wolbachia in two pathogen vector insects, Asian citrus psyllid and Longan psyllid
Source: Front Cell Infect Microbiol. 2023 Mar 6;13:1121186. doi: 10.3389/fcimb.2023.1121186 (PMC10025399; doi:10.3389/fcimb.2023.1121186)

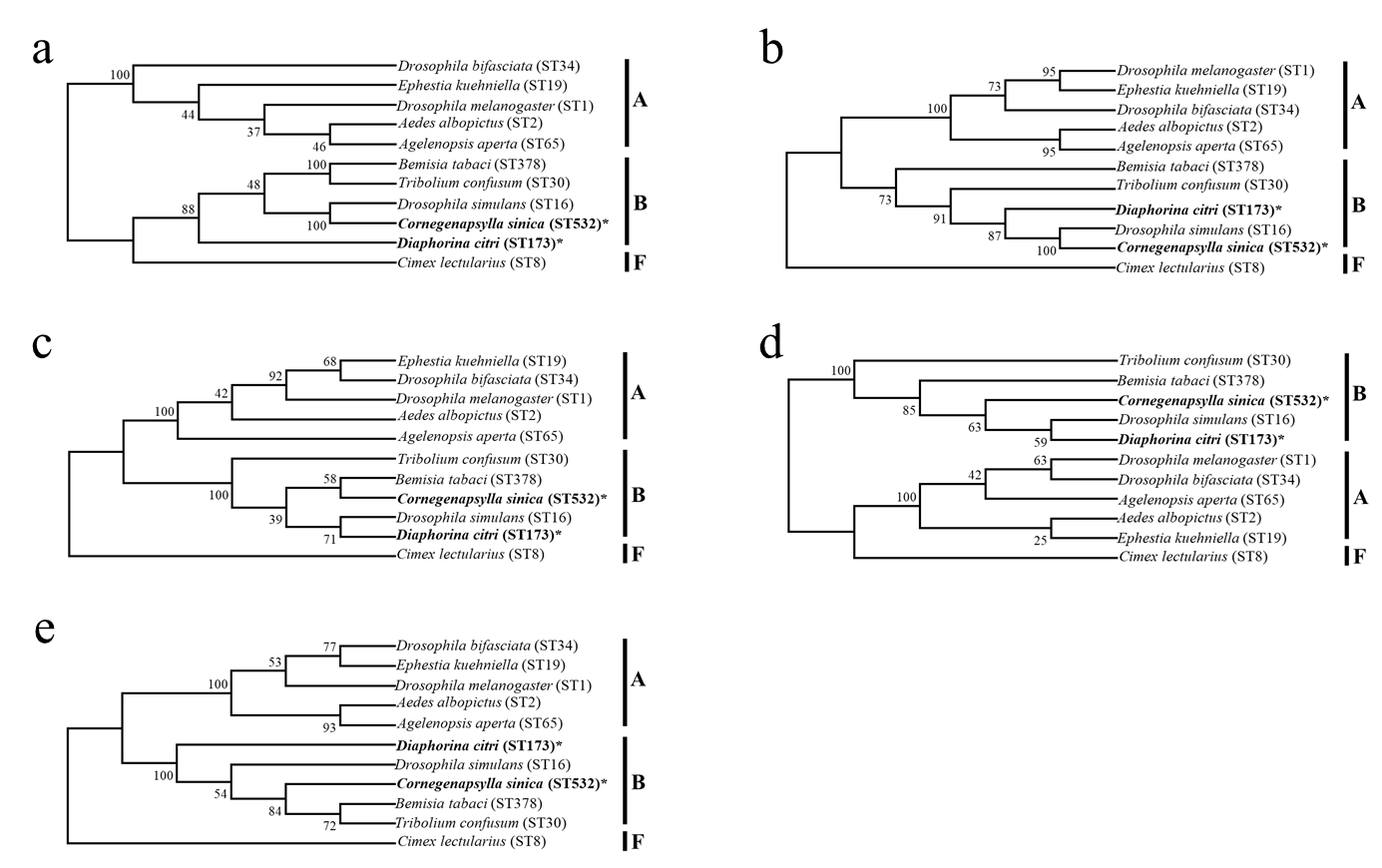

Supplement: Supplementary Figure 1 — The phylogenetic relationships of Wolbachia from different insect hosts based on the DNA sequence of (A) coxA, (B) fbpA, (C) ftsZ, (D) gatB and (E) hcpA gene. The tree was constructed and analyzed by Neighbor-Joining (NJ) method using 1000 bootstraps replicates. [file Image_1.tif]
